# Predictors of poor adherence in schizophrenia

**DOI:** 10.1192/j.eurpsy.2021.1415

**Published:** 2021-08-13

**Authors:** W. Bouali, R. Gniwa Omezzine, R. Ben Soussia, S. Younes, L. Zarrouk

**Affiliations:** 1 Department Of Psychiatry, University Hospital Of Mahdia, Tunisia., Psychiatry, Mahdia, Tunisia; 2 Department Of Family Medicine. Tunisia., Monastir Faculty of Medicine, Mahdia, Tunisia

**Keywords:** Schizophrenia, Antipsychotic drugs, Adherence, Non-adherence

## Abstract

**Introduction:**

Schizophrenia is a chronic mental disorder that requires long-term treatment. Non-adherence to antipsychotics is common and associated with poor outcomes.

**Objectives:**

Our study is aimed to describe the therapeutic adherence and to identify the factors associated with poor adherence among schizophrenic patients.

**Methods:**

This was a cross-sectional study conducted at psychiatry consultation of the university medical center of Mahdia, Tunisia. Data collection occurred between the months of January and March 2018, including patients suffering from schizophrenia. The evaluation of adherence was performed using the MARS scale (Medication Adherence Rating Scale).

**Results:**

In our sample of 131 schizophrenic patients, there is a male predominance (76%), as well as unmarried status (58.7%), unemployed (72%). The rate of non compliance treatment was 73%. Low levels of education, poor insight and polytherapy were associated to poor adherence. Although patients aged more than 40 years, who were married and diagnosed with undifferentiated schizophrenia were good compliant to treatment (p<0.05).

**Conclusions:**

We suggest a proper treatment strategy for each patient based on the identification of non adherence risk factors.

